# Virus Variability and Its Impact on HIV and Hepatitis Therapy

**DOI:** 10.1155/2012/607527

**Published:** 2012-12-27

**Authors:** Domenico Genovese, Christoph Boesecke, Nicola Coppola, Stefano Vella

**Affiliations:** ^1^Department of Therapeutic Research and Medicine Evaluation, Italian National Institute of Health (ISS), Rome, Italy; ^2^Department of Internal Medicine, University of Bonn, Bonn, Germany; ^3^Department of Public Medicine, Section of Infectious Disease, Second University of Naples, Naples, Italy

Human immunodeficiency virus (HIV) and hepatitis C virus (HCV) RNA genome, as well as hepatitis B virus (HBV) DNA genome, introduce several nucleotide substitution due both to the lack of proof reading activity of viral polymerase and to the high replication activity. As a consequence, viral populations are extremely heterogeneous and have been labeled molecular quasispecies. The virus population structure has numerous implications for the biology and associated pathology. This variability could be utilized by the virus to adapt itself to the different host conditions as immunitary system efficiency and presence or absence of antiviral therapy.

The knowledge of viral population variability molecular markers could be utilized to modify the classic approach to antiviral therapy based on generalized protocols. From such studies, it will become possible to estimate genetic diversity within a virus and its known strains, estimate patterns and rates of mutation for each viral gene or noncoding region, identify causal strains associated with pathological features and with specific antiviral response.

Due to their shared routes of transmission, hepatitis viruses infection is common among persons infected with HIV. Chronic coinfection with hepatitis C virus (HCV) is documented in one-third of all HIV-infected persons in the United States, especially in those with a history of intravenous drug use; it is associated with an increased liver-diseases-related morbidity and mortality compared with HCV or HIV monoinfection due to the prolonged HIV survival with the advances in ART therapy and to a more severe liver disease.

This special issue addressed a variety of aspects of the research against HCV and HIV infection with particular regard to specific aspects of the viral variability in relation to treatment and to the response of the immune system.

From 10 submissions, 7 papers are published in this special issue. Each was reviewed by at least two reviewers and revised according to review comments. The papers cover the following arguments: impact of HIV-1 genetic variation on AIDS; role of antibodies in the development of HIV infection outcome; immunology of HIV and HCV; role of HCV variability in therapy.

HIV-1 encodes a transactivating regulatory protein (Tat), which is essential for efficient transcription of the viral genome. Tat acts by binding to an RNA stem-loop structure, the trans-activating response element (TAR), found at the 5′-ends of nascent HIV-1 transcripts, recruiting a positive transcription elongation complex and increasing the production of full-length viral RNA. Tat protein also associates with RNA polymerase II complexes during early transcription elongation. All of these studies have motivated a number of researchers to use Tat as an important target in combination antiretroviral therapies, but to date none of these have materialized into clinically effective antiviral agents. The paper of L. Li et al. describes the different functions of this intriguing protein observing that the genetic variability within the Tat gene may impact the molecular architecture of functional domains of the protein and as a consequence afflict HIV pathogenesis and disease. Tat as a therapeutic target for anti-HIV drugs has also been discussed. The isolation of mutant sequences of Tat from different sites can also be used to identify tissue-specific functions of Tat that may have great bearing on long-term use of Tat inhibitors. We must also consider that the eradication of latent reservoirs by irreversibly blocking LTR activation or Tat transactivation activity could be a major step in controlling the HIV-1 pandemic.

The strains of HIV-1 can be classified into four groups: the “major” group M, the “outlier” group O, and two new groups, N and P. Within group M, there are known to be at least nine genetically distinct subtypes (or clades) of HIV-1. These are subtypes A, B, C, D, F, G, H, J, and K. Occasionally, two viruses of different subtypes can meet in the cell of an infected person and mix together their genetic material to create a new hybrid virus. Many of these new strains do not survive for long, but those that infect more than one person are known as “circulating recombinant forms” or CRFs. The classification of HIV strains into subtypes and CRFs is a complex issue and the definitions are subject to change as new discoveries are made. In the paper of R. Sampathkomar et al. the origin and development of quasispecies and CRFs has been deeply described as well as the role of antiviral and immunological response in HIV genetic makeup and the ability of host genetic factors to influence HIV-1 evolution. 

Integrase inhibitors are a class of antiretroviral drug designed to block the action of integrase, a viral enzyme that inserts the viral genome into the DNA of the host cell. Since integration is a vital step in retroviral replication, blocking it can halt further spread of the virus. The paper of X. Ni et al. describes the susceptibility of different HIV-1 strains to integrase inhibitors. This paper demonstrates by in-silico and in-vitro studies that the differences in the sequences of integrase gene from different subtypes do not affect the susceptibility to integrase inhibitors.

The V3 loop of the HIV-1gp120 glycoprotein presenting 35-residue-long, frequently glycosylated, highly variable, and disulfide bonded structure plays the central role in the virus biology and forms the principal target for neutralizing antibodies and the major viral determinant for coreceptor binding. HIV-1's subtype C V3 loop consensus sequence exhibits increased resistance to anti-V3 antibody-mediated neutralization as compared to the subtype B consensus sequence. D. Almond et al. in their paper describe the differences in the three-dimensional V3 loop structures between subtypes B and C. Differences in the flexibility in specific *β*-strand structure could be responsible for the diverse binding activity of anti-V3 antibody. The different susceptibility to human antibody-mediated neutralization could also result in different extents of global spread between different subtypes.

Effective immunity to HIV is poorly understood. Cytotoxic T lymphocytes and neutralizing antibodies readily select for immune escape HIV variants. It is now also clear that NK cells can also select for immune escape through both direct Killer-immunoglobulin-like-receptor- (KIR-) mediated killing as well as through facilitating antibody-dependent cellular cytotoxicity (ADCC). Viral fitness is a complex parameter illustrating the overall contribution of all mutation-related advantages and losses. Even though the evasion of immune responses presented by escape mutations presents a definite fitness benefit to the virus, the HIV-1 proteome is not infinitely malleable hence the same mutations can result in fitness costs. Some immune escape variants have reduced replicative capacity of the virus (reduced “fitness”) that slows the progression of disease. There is an urgent need to identify effective immunity to HIV and to use this information to design efficacious vaccines and immunotherapies, and the paper of Isitman et al., contributes efficiently to this knowledge, reviewing the introduction and effects of immune escape driven by CTL, neutralizing antibodies and NK cells. 

Viral immunology is a rapidly evolving field. Major strides have been made in our understanding of innate and adaptive immune responses to viruses, largely based on highly reductionistic animal infection models, but more recently in humans, with validation that fundamental immunological concepts do in fact translate into clinical science well. From these studies has been emerging an appreciation of the enormous complexity of the immune response to viral infections as well as the diverse array of strategies developed by viruses to deal with immune detection. HIV and HCV markedly differ in their virological properties and in their pathogenesis but share many common features in their immune escape and survival strategy. Both viruses have developed sophisticated ways to subvert and antagonize host innate and adaptive immune responses. In their interesting paper M. G. Quaranta et al. describe current knowledge on innate and adaptive immune recognition and activation during HIV and HCV monoinfections and evasion strategies as well as the genetic associations between components of the immune system, the course of infection, and the outcome of the therapies.

HCV infection therapy is based on a combination of pegylated interferon and ribavirin. This therapy is, unfortunately, very little effective (only a percentage of around 50% of the subjects respond to drugs and, moreover, a part of these is subsequently reinfected with the virus) and is associated with major side effects. For this reason, taking into account the experience with the anti-HIV therapy, new HCV direct antiviral drugs (STAT-C, including protease inhibitors and polymerase inhibitors) are already at an advanced stage of experimentation. Telaprevir and Boceprevir (two protease inhibitors) have been introduced in the USA guidelines for HCV genotype 1 therapy. The new therapeutic options are able to significantly increase the sustained virological response (SVR) compared to the current standard therapy, and potentially reducing the overall duration of the treatment. However, the occurrence of side effects requiring discontinuation of treatment makes it critical to optimize the therapy. In their paper, C. Strahotin and M. Babich summarize the current knowledge on the pathogenesis of HCV therapy with particular reference to therapy and introduce the problem of resistance to the new protease inhibitors. This is a highly relevant and interesting topic because new upcoming compounds are likely to be much more effective than the present. Probably, a highly effective interferon-free regimen for the treatment of HCV infection could be soon available, and the problem of resistant mutants could be a focal point of anti-HCV therapy research.

These papers represent an exciting, insightful observation into the state of the art, as well as emerging future topics in a moment when strategies for the fight against HIV and HCV are at a critical point. For HIV we start to talk about HIV eradication strategies, while the development of new HCV therapies, for the first time, gives us the opportunity to finally defeat this disease and its important and serious sequelae. We hope that this special issue would attract a major attention of the peers. We would like to express our appreciation to all the authors and reviewers.

*Domenico Genovese* *Christoph Boesecke* *Nicola Coppola* *Stefano Vella*

